# To Use or Not to Use – Practitioners’ Perceptions of an Open Web Portal for Young Patients With Diabetes

**DOI:** 10.2196/jmir.1987

**Published:** 2012-11-09

**Authors:** Sam Nordfeldt, Teresia Ängarne-Lindberg, Carina Berterö

**Affiliations:** ^1^Division of Child and Adolescent PsychiatryDepartment of Clinical and Experimental MedicineLinköping UniversityLinköpingSweden; ^2^Center for Medical Technology AssessmentDepartment of Medicine and Health SciencesLinköping UniversityLinköpiingSweden; ^3^Division of Nursing SciencesDepartment of Medical and Health SciencesLinköping UniversityLinköpingSweden

**Keywords:** Internet, Web 2.0, Health knowledge, childhood chronic disease, health professionals, practice, attitudes, participation, type 1 diabetes, health information, blog, discussion forum

## Abstract

**Background:**

Health care professionals' attitudes can be a significant factor in their acceptance and efficient use of information technology, so they need to have more knowledge about this resource to enhance their participation.

**Objective:**

We explored practitioners’ perceptions of using an open-access interactive Web portal tailored to young diabetes type 1 patients and their guardians or significant others. The portal offered discussion forums, blog tools, self-care and treatment information, research updates, and news from local practitioners.

**Methods:**

Eighteen professionals who were on pediatric diabetes care teams each wrote an essay on their experience using the portal. For their essays, they were asked to describe two situations, focusing on positive and negative user experiences. The essays were analyzed using qualitative content analysis.

**Results:**

Based on our analysis of the respondents essays, we identified three categories that describe perceptions of the Web portal. The first category - to use or not to use - included the different perspectives of the practioners; those who questioned the benefits of using the Web portal or showed some resistance to using it. The frequency of use among the practitioners varied greatly. Some practitioners never used it, while others used it on a daily basis and regularly promoted it to their patients. Some respondents in this category reflected on the benefits of contributing actively to online dialogues. In the second category - information center for everyone – practitioners embraced the site as a resource for scientifically sound information and advice. As part of their practice, and as a complement to traditional care, practitioners in this category described sending information through the portal to patients and their significant others. Practitioners felt safe recommending the site because they knew that the information provided was generated by other practitioners. They also assumed that their patients benefited from actively using the Web portal at home: peers brought the site to life by exchanging experiences through the discussion forums. In the third category – developing our practice – practitioners reflected upon the types of information that should be given to patients and how to give it (ie, during in-person appointments or through the Web portal). They perceived meeting with various professionals at other hospitals to update information on the portal and develop content policies as constructive teamwork. Practitioners expressed interest in reading patients’ dialogues online to learn more about their views. They also thought about how they could use the portal to adapt more to patients’ needs (eg, creating functions so patients could chat with the diabetes nurses and doctors).

**Conclusions:**

Practitioners expressed positive perceptions toward a tailored open Web portal. They suggested that future benefits could be derived from systems that integrate factual information and online dialogues between practitioners and patients (ie, exchanging information for everyone’s benefit).

## Introduction

Life with pediatric diabetes type 1 can be a more or less daily struggle that involves extensive self-care and a constant interplay between patients and others involved in their care [1]. Coping skills are essential because management of the disease, including insulin injections and self-control of blood glucose, affects everyday life. Most of the treatment is performed by the young patients themselves and their guardians or significant others. Over time, they become their own experts [1], which means that they must keep themselves up to date about treatment, self-care, and scientific findings. They are also guided through the varying phases of the disease trajectory by multi-professional pediatric diabetes teams. For patients to effectively manage their life with diabetes and minimize possible short- or long-term complications, they require guidance and supervision from practitioners [1-4]. Effective physician-patient communication has long been shown to be important in patient satisfaction, treatment adherence, and health outcomes [5-9].

 In the context of self-management education, the Internet is a dynamic and promising resource that opens new ways for both medical practitioners and patients to communicate with each other and educate themselves [10-12]. Nevertheless, some practitioners have expressed doubt about introducing the new technology into their practice. Although practitioners may recognize the general benefits, uptake of Web-based technologies has been slow [13]. Studies have identified lack of access, lack of time, and lack of opportunities for training as examples of causal factors preventing practitioners from adopting new technologies [14]. Few practitioners were familiar with the rapidly emerging social networking tools on the Internet and, for some, patient access to electronic information and communication may even have been a source of irritation [10, 12]. To some extent, practitioners’ negative attitudes toward new technologies have been related to factors such as age and time since completion of education [14-15].

 Other practitioners found that using Web portals in routine care facilitated their work because the sites saved them time and simplified their routines [16-17]. However, for practitioners to benefit from using the Internet in health care, they need to increase their own involvement with and skills in using this technology [18-20]. A recent review indicates that practitioner involvement is a significant factor in patient acceptance and efficiency of Internet use; thus, efforts are needed to enhance practitioners’ participation in Web-based technologies [20].

Perception refers to the knowledge gained from a process of coming to know or understand something. This implies the ability to understand inner qualities and relationships. While many studies focus on how patients perceive and use Web portals, little is known about practitioners’ perceptions of using such technology for dialogues with and among patients.

We aimed to explore practitioners’ perceptions of an open Web portal tailored to young diabetes type 1 patients as well as their guardians, school staff, and significant others.

## Methods

**Process of Care**

In Sweden, children and adolescents with diabetes are treated by hospital-based pediatric diabetes teams that may include nurses and nurse specialists, physicians, dietitians, social workers, and clinical psychologists. These teams educate, advise, and support patients, families, and significant others through the course of a complicated treatment. Typically practitioners meet the young patients, along with their guardians, when patients are hospitalized at onset. Practitioners continue to see their patients every three months or more often as outpatients over many years.

Practitioners and patients usually communicate through regular appointments in the clinic and telephone contact when needed. Local websites of some health care providers include basic patient information and links to other websites. Electronic communication systems are increasingly available, but patient and provider uptake has been slow.

### Web Portal

In November 2008 the Diabit LIST research group and two participating pediatric diabetes teams launched an open-access Web portal for young diabetes type 1 patients and guardians, school staff, and significant others. The objective of the portal was to complement traditional treatment by enhancing diabetes-related information for and communication with and among young diabetes type 1 patients. The portal provided access to extensive general, as well as local, information. It also provided peer-mediated information and dialogues through open-access forums and blogs [12, 21]. The medical information provided on the website was generated by experienced practitioners. Both patients and practitioners contributed to the portal as forum moderators.

**Figure 1:**
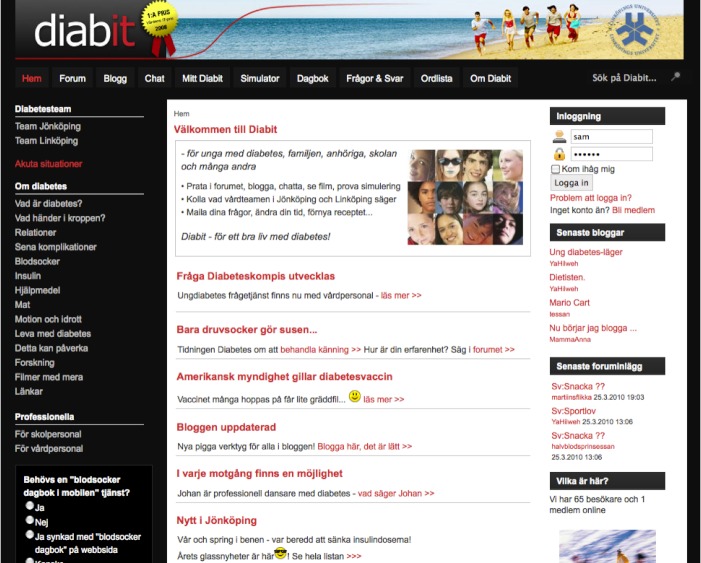
Screenshot of welcome and news feed page, which includes “About Diabetes,” “Diabetes Teams,” “Latest Blogs,” “Latest Forum Posts,” “Who Is Here?,” “Questions and Answers,” “Word List,” and “Search.” Registration and login were required only for active contribution to portal content (eg, blogs and discussion forums).

**Figure 2:**
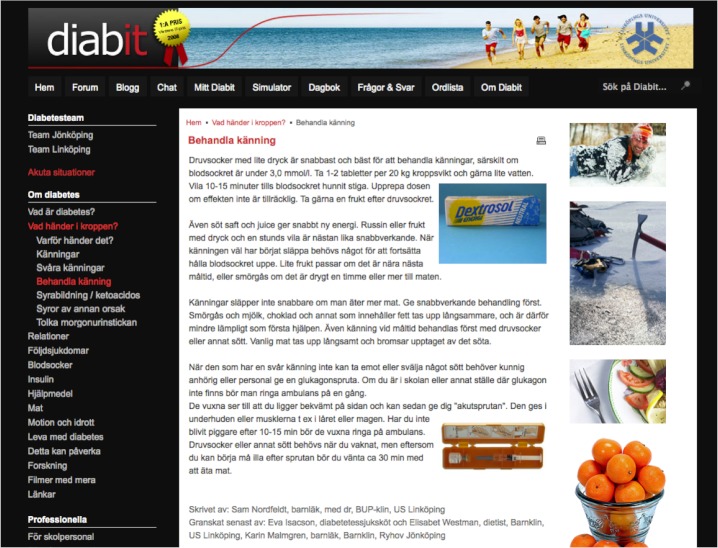
Screenshot of sample fact page: About Diabetes/What happens in the body?/Treating hypos.

**Figure 3:**
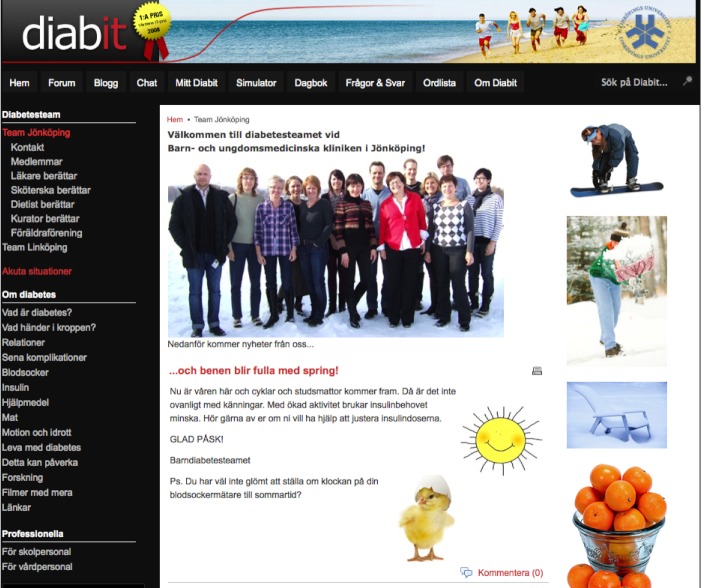
Screenshot of a local diabetes care team’s welcome and news page, menu with contact information, and staff information.

**Figure 4:**
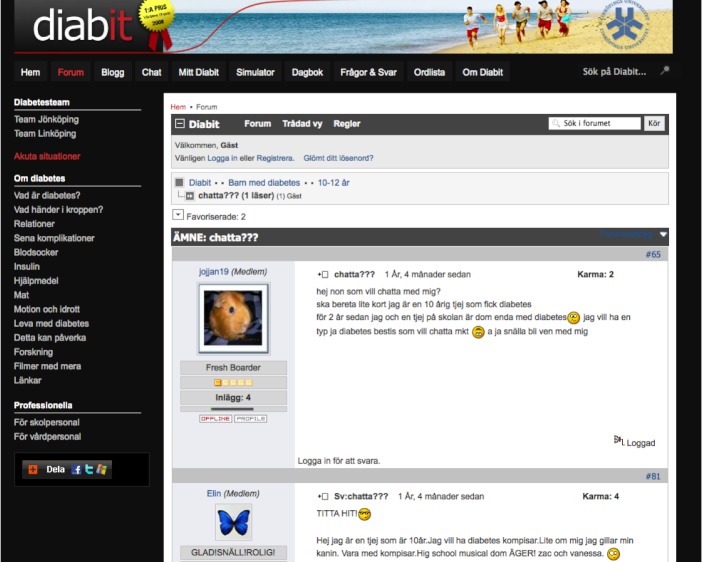
Screenshot of forum posts from patients in the 10-12 years age group.

The portal offered open discussion forums for children, adolescents, and guardians. There were blog tools so anyone could post a blog, and there were also links to the blogs created by patients with diabetes and guardians. It also provided comprehensive self-care and treatment information from practitioners, including advice for specific situations, as well as feeds that supplied research updates and news about devices, products, and activities (Figures 1-4). In addition, a member of each clinical diabetes team managed a local news feed that was integrated in the portal. These information sites included local contact information, staff presentations, and reciprocal services for prescription renewal, booking appointments, and text message exchange between patients with guardians and practitioners. To our knowledge, this portal design remains unique in pediatric care [12, 21].

The portal was gradually developed through a user-centered design process that included iterative sessions conducted over a number of years with groups of patients, guardians, and practitioners [12]. An early interview study found that participating practitioners, regardless of their professional roles, had positive attitudes toward the portal [12].

Local practitioners participated in the portal as part of routine care. After the launch of the open portal, practitioners slowly increased their involvement. Their participation evolved into extra voluntary personal commitments as well; some practitioners edited local news feeds or monitored patient forums. Biannual collaborative group activities included the different professionals updating information on the portal, holding policy discussions, and setting goals for the use and continuous development of the portal.

### Sample and Data Collection

In May 2010, 24 practitioners were invited by email (with 2 reminders) to write an essay describing their experience using the portal. The invitation letter asked participants to focus on situations that represented positive and negative user experiences. Clarifying questions were provided to help practitioners start their essay writing:

(1) Describe a situation when you succeeded in using [the site]. Has [the site] made things easier for you in any way? Are there any advantages in using [the site]? Describe possibilities for using it.

(2) Describe a situation when you did not succeed in using [the site]. Has [the site] become an obstacle in some way? Are there any disadvantages in using [the site]? Describe any obstacles in using it.

Of the 24 practitioners who received the invitation by email, 18 people (members of 2 pediatric diabetes care teams, including doctors, nurses, dietitians, and a social welfare officer) wrote an essay.

### Analysis

The 18 essays were analyzed using modern techniques of qualitative content analysis. This method can be applied to transcribed interviews, texts, and narratives, for example [22, 23]. Qualitative content analysis allows the study of both apparent and latent content, which, in turn, allows the tracking of emerging or new perceptions.

Initially, two researchers (SN and CB) independently read and analyzed the essays. Statements with similarities were clustered and summarized into tentative themes based on their emerging contents. The tentative themes with all respective statements were reviewed in detail. Unclear statements were explored with respect to the original context. Before open comparisons, both of them again read all the primary data and the material emerging in the analysis. Through iterative in-depth discussion sessions, a stepwise re-categorization and repeated validation against the complete original essays was performed. A more logical and complete structure consisting of three categories gradually emerged. Any discrepancies were resolved through discussion; no measure of inter-rater reliability was used. Both apparent and latent content was considered important [22].

## Results

The results are presented as 3 categories that we identified and interpreted as responses that described situations representing positive and negative user experiences and answered the supporting questions. The category “to use or not to use” was built up from statements related to whether or not respondents found the platform useful in their practice. The second category, “information center for everyone,” represents respondents who embraced the portal as a source of scientifically sound information and advice for patients, guardians, and significant others, as well as for other professionals. The final category, “developing our practice,” includes respondents that reflected on what information to give through the portal and how to give it, learning more about patients’ views, and adapting more to patients’ needs.

We include quotations from individual essays within the descriptions below to confirm and illustrate each category .

### To Use or Not to Use

The frequency of use among the practitioners varied greatly. Some practitioners never used the Web portal, while others used it on a daily basis and regularly promoted it to their patients. Many respondents stated that they visited the website now and then, just to look around. Many felt it was worthwhile using it in the clinic and recommended it to patients. Some also reflected on the idea of contributing actively to online dialogues themselves.

Sometimes I read facts, alone or with patients. I also read blogs, but don’t post anything myself.

There were positive statements concerning the existence, design, and function of the website. Overall, respondents thought that it mostly functioned well. Users considered the design easy to understand. Practitioners found it to be a manageable tool when seeking information. The information was easily accessible to everyone: wherever there was a computer, the information was close by.

The advantages are that it is handy if you need to look something up, accessible information, and that it is easy to search.

The problems that respondents perceived when using the website comprised a range of factors, such as technical problems, lack of time, inadequate computer experience, a lack of commitment, a lack of computer access, and disorganized information.

I think that [the site] is very messy; it is not orderly enough and there is almost too much information in one place, which makes it hard to find what I want to read about…

### Information Center for Everyone

Respondents in this category viewed the Web portal as a source of scientifically sound information and advice that is available to several categories of professionals, as well as patients and their significant others. As a complement to traditional care, practitioners described various situations in which they mediated information through the portal to patients and their significant others. Some practitioners demonstrated how to use the website as part of their practice, while others did not.

I have never received a “no” when I’ve asked if I can show the site.

Some practitioners also assumed that their patients benefited from actively using the website at home: as peers exchange experiences, the site comes alive. Practitioners believed that the portal would help patients and their families learn more at home, enabling them to manage various situations themselves. Answers to questions could be found on the website without patients having to make a call to the pediatric diabetes care team.

The big patient benefit is if many patients are there so that they can exchange their different experiences, which makes the site come alive…

Although the information was targeted to practitioners, patients, and significant others, easy access to the website made it useful for everyone, including new staff, students, school and preschool staff, primary-care and other hospital staff.

I recommend [the site] to all patients, parents, and other relatives. I also recommend it for new and old staff. School and preschool staff can visit the site to prepare themselves prior to our visits.

Practitioners felt safe in recommending the website because they knew that the information was produced by, and the practitioners were part of, a multi-professional community. Provided that all practitioners adhered to the same facts, the information presented on the site would be consistent.

What feels safe for me as a part of the nursing staff is that the information they can read here is the information we have provided. We know that we have critically reviewed it together.

### Developing Our Practice

 Respondents in this category include those who reflected on what information to give and how to give it (eg, during in-person appointments or through the Web portal). Practitioners perceived updating the information, as well as the content policies, in multi-professional meetings with other hospitals as constructive teamwork.

It is useful to have discussions and to hear what other people think concerning diabetes treatment.

Use of the website increased over time. Over the course of the project, more team members contributed new information and updates to the local editors, and practitioners referred patients to content on the website during clinical visits.

For example, they have read about a lecture, tried a recipe, or printed out facts or advice from the site.

Practitioners expressed interest in adapting their practice to better meet patients’ needs, including new services on the Internet. They also indicated they would like to learn more about patients’ views by reading dialogues online. Some practitioners found it advantageous to see what questions patients asked and what answers they got.

I also follow forums and the blogs without posting anything myself. By doing so I learn how the patients think, which can be useful in working with them.

Individual respondents also reflected on whether increasing online public exposure to differences between hospitals’ policies (eg, differing policies for the use of technical devices) implied difficulties, or if it actually enhanced constructive dialogues.

If we collaborate with [another clinic] that is much more generous than we are, with [insulin] pumps for example, it might be more difficult. Or would this become a constructive dialogue? Online, the clinic is a little more public.

Assuming that they would communicate with patients and their significant others online to an increasing extent, practitioners suggested ways of adapting the site to better meet their patients’ needs. Ideas included using more functions on the site that their patients need, referring to preparation sheets before clinical visits, developing prescription and appointment-booking services, providing more frequent updates on food and nutrition, making the site easy to find through search engines, and introducing online chats with nurses and doctors.

For example, chatting in the future with the diabetes nurses or the doctors.

## Discussion

The present study demonstrates positive perceptions among health care practitioners regarding the development and use of a Web portal in their practice. Most of the participants wrote in a positive way about the website’s existence, design, and function. They pointed out that the site represented scientifically sound information and tips that were useful for several categories of professionals and patients. Although most respondents felt it was worthwhile to use the website in the clinic and recommended that patients should use it, some expressed resistance to using it.

### To Use or Not to Use

 Use of the Internet as a resource and a means for improving health and health care has attracted considerable attention, but success in adopting these communication technologies depends on the degree of acceptance by its users [24-26]. In light of the important role practitioners play in supporting self-care management, use of a Web portal among patients requires that practitioners actively use it and advocate its use to their patients [21]. We found resistance due to a lack of time, inadequate familiarity with computers, lack of commitment, lack of computer access, and disorganized information on the site. Our findings support previously reported causes for resistance and limited use of new technologies, including little training in computer use in basic education or professional life, a lack of time and opportunities for training, and a lack of access to the technology [14, 19].

The goal of developing health information portals is to produce health benefits for patients with long-term illness. As such, health care practitioners are essential intermediaries of knowledge delivered through these portals. Therefore a process enabling practitioners to develop the skills necessary to participate in portal design and content development is needed. We previously found that participation in portal development could produce a reciprocal willingness to integrate its use into routine care [12]. Hence practitioners are more willing to use and promote health information portals if they perceive that they gain knowledge by developing and using content on the site and thus understand its usefulness. In addition, practitioners will develop new skills and their work will be facilitated due to time-savings and simplified routines [12]. It is possible that the respondents perceived participation in the present study as a meaningful use of already acquired knowledge in a new arena. Clearly, efforts are needed to offer local practitioners a comfortable and time-efficient process for participation.

### Information Center for Everyone

Internet use has the potential to improve access to health care by removing barriers associated with physical location and the need for improved communication [25, 27-28]. For instance, the wider implications of certain topics may not become apparent to patients until they have had some time to reflect; once they process the information new questions may arise. It is not unusual for patients to feel frustrated because their needs are not fully met during the course of medical encounters [27]. Therefore, it is not surprising that practitioners viewed the portal in this study as a valuable complement to traditional care. They also perceived it as a source of knowledge for patients and their significant others, as well as for health care professionals (ie, as an information center for everyone). Respondents perceived several advantages to using the Web portal, including providing support for patients to learn more at home, providing easily accessible information, and being useful for a range of people such as school and preschool staff, primary-care practitioners and colleagues in other hospitals, as well new health care staff and students.

The practitioners required that the information given on the portal be trustworthy. In earlier studies, trustworthiness has been described as the absence of commercial interests and up-to-date information, including clear references for the information given [29-30]. In our study, respondents noted that knowing that practitioners and their colleagues were the authors signified a new dimension of trustworthiness. Participation in creating the content on the website made practitioners feel secure in recommending the portal to patients, their guardians, and significant others, as well as referring other professionals (such as preschool and school staff) to the portal. As reported earlier, since the information on the portal was identical to the information provided at the clinic, it was perceived as congruent, familiar, and appropriate for patient use [12]. This appears to be a practitioner-derived dimension of trustworthiness based not only on scientific knowledge and evidence, but also on their participation and collaboration in developing content for the site through multi-professional teamwork.

### Developing Our Practice

As use of the Internet leads to a shift in the role of patients from passive recipients to active consumers of health information, practitioners face an increasing demand that they be familiar with a variety of reliable, high-quality sources of online health information and medical websites [31]. The practitioners noted that they needed to collaborate and coordinate information not only between clinics, but also between clinics and the portal. Through this process, practitioners reflected on what information to give and how to give it. Taking advantage of the clinical experience and knowledge of many practitioners to update content on the portal represents a new development in the emerging practices of health portals. Thus our study suggests that practitioners should be involved in developing up-to-date health portal functionality and design solutions, updating information on the site, participating in online dialogues, and advocating the portal to their patients.

Effective care requires focusing attention on both the diseases themselves and patients’ experiences of their illnesses. The disease is what is wrong with the body. Illness is the patient’s personal experience of the disease, such as their thoughts, feelings, and altered behaviours [32]. The portal offers a process for practitioners, patients, and significant others to learn about the disease as well as the illness, particularly through users’ participation in discussions, blogs, and questions and answers posted on the forum. Such new experiences are useful for developing the practitioners’ perspectives on their practice and how they might better adapt to patients’ needs. Hence use of the Internet is not to be seen by patients as a replacement for practitioners and interpersonal meetings, but rather as a complement to traditional care [31]. The Internet offers new knowledge for practitioners, patients, and significant others.

### Limitations of the Study

In this study, qualitative methods were used to gain a deeper understanding of the respondents’ perceptions, so it is not possible to make generalizations in a quantitative manner [33]. The essays were a rich source of information even though they were rather short. It is possible that some way of giving respondents a dedicated time for the task would have further expanded the amount of data obtained. We can also speculate that some non-responders (and, perhaps, some responders), felt uncomfortable writing an essay, did not feel very familiar with the portal, or were not particularly motivated or interested in the topic.

### Conclusions

The results of this study suggest that there is value in having practitioners continuously involved in developing online content and participating in dialogues with and between patients. The practitioners’ positive perceptions of the portal as a new tool in their practice might be an early indication of a forthcoming change. Their perception of the site’s trustworthiness includes not only scientifically sound information but also their own participation in creating online information to support their patients. Indeed, the findings suggest future benefits from systems that integrate factual information with open dialogues between local practitioners and their patients, or an exchange of information for the benefit of everyone.
